# Envelope Glycoprotein Trimers as HIV-1 Vaccine Immunogens

**DOI:** 10.3390/vaccines1040497

**Published:** 2013-10-28

**Authors:** Quentin J. Sattentau

**Affiliations:** The Sir William Dunn School of Pathology, The University of Oxford, South Parks Road, Oxford OX13RE, UK; E-Mail: quentin.sattentau@path.ox.ac.uk

**Keywords:** HIV-1, vaccine, neutralizing antibodies, envelope glycoproteins, trimer

## Abstract

The HIV-1 envelope glycoprotein spike is the target of neutralizing antibody attack, and hence represents the only relevant viral antigen for antibody-based vaccine design. Various approaches have been attempted to recapitulate Env in membrane-anchored and soluble forms, and these will be discussed here in the context of recent successes and challenges still to be overcome.

## 1. Env as a Viral Entry Machine

**Figure 1:**
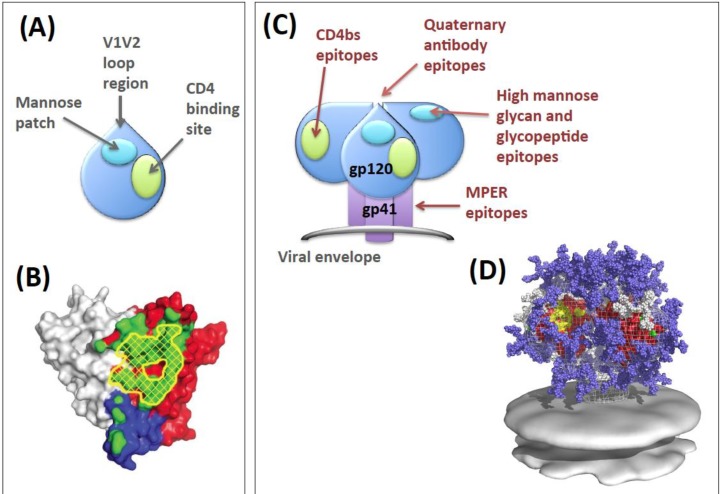
HIV-1 Env. (**A**) Cartoon of gp120 with major features represented. The location of the V1V2 loops, missing from gp120 crystal structures, is predicted from analysis of trimeric Env by electron tomography [10,11] and from the location of the quaternary conformation epitope-specific antibody PG9 by negative stain electron microscopy [12]; (**B**) Molecular model of gp120 based on crystal structures and obtained with permission from [13]. The gp120 surface is colored grey for inner domain, red for outer domain and blue for the bridging sheet. The initial contact surface for CD4 is shown in yellow cross-hatching, and the recognition surface of broadly neutralizing CD4bs antibody VRC01 is green; (**C**) Cartoon of the Env trimer with broadly neutralizing antibody epitopes depicted; (**D**) Molecular model of Env trimer with glycans. Red surface is gp120 density, yellow represents the CD4 binding site, hybrid and complex glycans are represented in blue and the 2G12 epitope high mannose glycans in white (from [14]).

To fully grasp the potential of the HIV-1 Env trimer for vaccine use, its structure and function need to be understood. HIV-1 carries a single virally-encoded structure on the outer surface of its envelope, the Envelope glycoprotein (Env). Env consists of a non-covalently linked trimer of heterodimers, each heterodimer composed of one surface gp120 subunit and one transmembrane subunit (Figure 1). The *env* open reading frame codes for a precursor trimer polypeptide (gp160) that is trafficked from the endoplasmic reticulum to the Golgi, within which it is cleaved by a cellular protease into its mature components [1]. Cleavage refolds Env into an activated, fusion-competent state via undefined structural rearrangements within the trimer. The mature trimer is then trafficked to the plasma membrane via a poorly defined pathway [1] that may involve regulated secretion induced by contact of the infected cells with an uninfected, receptor-bearing cell [2]. The cytoplasmic tail of gp41 carries endocytic motifs that drive traffic Env from the plasma membrane into either maturing endosomes leading to degradation or recycling endosomes, meaning that at steady state a large proportion of Env is within intracellular compartments [3,4]. This is suggested to be an immune evasion strategy, reducing cell surface Env recognition by B cells. Env targets lipid rafts via an acylation signal on gp41, meeting Gag at the cell membrane to initiate budding of the nascent virion [1]. The surface location of Env is dependent upon the infected cell type: CD4^+^ T cells predominantly express Env at the plasma membrane, whereas macrophages express Env principally within an intracellular compartment continuous with the plasma membrane called the virus-containing compartment (VCC) [5,6]. 

The Env gp120 subunit is the receptor-binding component, and is comprised of a three-domain substructure: the inner and outer domains and the linking bridging sheet [7] (Figure 1). Gp120 engages CD4 and a coreceptor (CR), either one of the chemokine receptors CCR5 or CXCR4, in a two-step process. The CD4 binding surface (CD4bs) on gp120 is constitutively accessible to CD4 on Env, but undergoes a conformational change stabilized by CD4 binding that triggers both high-affinity CD4 binding and structural rearrangement of the trimer to reveal the chemokine-binding surface (CRbs) [8]. Subsequent CR engagement by gp120 leads to further conformational rearrangement of the Env trimer that triggers gp41 activation leading to its refolding and proposed penetration of the target cell membrane [8]. The formation of a coiled-coil gp41 structure brings the viral and target cell membranes into close apposition, driving their fusion and allowing entry of the viral core into the cell [9].

## 2. Env Antibody Evasion Strategies

Env is essential for viral infectivity, and antibodies targeting functional Env trimers will neutralize virus by preventing receptor engagement and/or virus-cell fusion, inhibiting both cell-free and cell-to-cell spread [15]. Non-neutralizing antibodies may also impact upon HIV-1 replication by binding non-functional Env on HIV-1-infected cells and mediating effector functions such as antibody-dependent cellular cytotoxicity, antibody-dependent cellular phagocytosis and IgA-mediated viral aggregation and sequestration in mucous [16,17,18]. Since the correlates of protection from immunodeficiency virus infection by neutralizing antibodies are robust [19,20,21,22,23,24], whereas those for non-neutralizing antibodies are not [25], here I will focus on neutralizing antibodies. HIV-1 has evolved a series of immune evasion strategies to reduce or abrogate the impact of neutralizing antibodies on replication. These have been extensively reviewed elsewhere [26,27,28], therefore the major mechanisms of antibody evasion by Env are only briefly, and non-exhaustively summarized. 

The primary immune evasion strategy concerns amino acid sequence variation in Env [29]. Although there is relative conservation of the receptor binding surfaces on gp120 and of several other regions of gp120 and gp41, major segments appear to be immunorecessive compared to the more variable portions of the molecule [27]. Thus the gp120 hypervariable regions, particularly the loop structures V1, V2 and V3, are primary antibody targets, but these antibodies can only neutralize a fraction of circulating viruses. Focusing the B cell response away from hypervariable regions and towards more conserved regions is a major challenge to antibody-based vaccine design.

Env is very heavily glycosylated, and glycan masking of the underlying protein surface prevents ready access of antibodies to vulnerable conserved Env regions [30]. A specific example of this is the CD4bs, where a “fence” of glycans reduces the ability of antibodies to probe the CD4bs in the same way as CD4 itself [31], resulting in antibodies that bind more weakly to Env and hence have limited neutralization activity [32]. However, recent crystallographic studies have revealed that glycans can also entirely comprise [33], or be a significant part [34,35,36], of neutralizing antibody epitopes, and so may in fact turn out to be part of the solution to the problem. 

A related accessibility issue concerns steric hindrance of antibody access to conserved neutralizing epitopes by structures other than glycans. The CD4bs forms a shallow valley on gp120 into which domain 1 of CD4 can fit snugly (a single immunoglobulin domain), but which an antibody variable region (two immunoglobulin domains) does not readily access [7]. A second level of steric exclusion is also exercised within the Env trimer since the gp120 protomers are arranged in a configuration that presents a very limited “angle of approach” to the CD4bs [37]. The element of the CRbs that is formed when gp120 adopts the CD4 bound state is also sterically restricted for antibody access when the Env spike is engaged with target cell CD4: there is insufficient space between the target cell membrane and the top of the Env trimer to accommodate the bulk of an IgG molecule [38]. Size restrictions may also apply to the accessibility of MPER antibodies to their epitopes, as they are wedged between the lower surface of gp120 and the viral envelope [39].

The trimer structure is reported to be metastable and able to adopt different conformational states, which are hypothesized to relate to an unliganded state, a CD4 bound, CRbs-accessible state, and an intermediate between these. Evidence for this comes from the ensemble of antigenic [40,41], functional [42], biochemical [43] and electron tomographic or single particle [44,45,46] analyses of different conformational states of intact trimers. Oscillation of Env between different conformational states is proposed to be part of a conformational barrier to antibody (and by extension B cell receptor) recognition [47]. 

An extreme example of conformational change comes from the dissociation of the trimer into subunits, by which gp120 is released in a soluble form [48,49] and gp41 adopts a post-fusion membrane-anchored conformation. This can be CD4-induced or spontaneous. Neither of these non-functional forms is likely to be able to induce potent or broad spectrum neutralizing antibodies. Env expressed at the plasma membrane of cells may be misfolded or uncleaved gp160, which are poor mimics of the functional trimer. Furthermore, HIV-1 infected cells may be lyzed by CTL or die by necrosis, releasing non-functional precursor forms of Env that will be recognized by B cells [50]. 

A final consideration relates to the number of Env spikes on a virion. This has been estimated to average approximately 10 [51,52] (although this number varies between different viral strains), a surface density that is suboptimal for cross-linking B cell receptors (BCRs) [53]. It has been hypothesized that the low number of spikes may lead B cells to undergo heteroligation (binding to two different epitope structures on an antigen) in order to drive high avidity antibody production [53]. 

## 3. Env as a Vaccine Antigen

When taken together, these evasion strategies provide a formidable barrier to antibody efficacy during natural infection, and make vaccine antigen design particularly difficult. However, results from diverse studies provide hope that trimeric Env, either native or structurally modified, might nevertheless be a useful antigen to elicit neutralizing antibodies in the appropriate immunization context. Historically, the first attempts at immunization with Env-based antigens used monomeric gp120 [54]. The neutralizing antibody responses obtained were relatively weak and were directed against a very limited spectrum of neutralization sensitive viral strains, termed tier-1 viruses, which do not represent most circulating strains [55]. It became apparent that the hypervariable V3 loop is a particularly immunodominant surface on gp120. However, despite efforts over 2 decades, V3 loop-based immunogens have failed to elicit antibodies that robustly neutralize a substantial proportion of circulating viruses that are intrinsically relatively neutralization resistant (termed tier 2) [56]. The most likely explanation for this is that the V3 loop is not well exposed on the majority of non-CD4-bound HIV-1 Envs, most likely as a result of packing into the surface volume of the trimer [57,58]. On soluble gp120 the V3 loop is liberated from constraints imposed within the trimer and becomes highly accessible to B cell recognition [59]. Analyses of antibody binding in sera from animals immunized with monomeric gp120 revealed strong binding to monomeric gp120 but very weak binding to trimeric Env [60]. This was one of the first indications that the immunogenic surfaces of gp120 that are exposed when the Env subunits dissociate are not represented on the trimer and are neutralization irrelevant. Together with the observed correlation between antibody binding to trimeric Env and neutralization [61,62], these findings prompted two considerations: firstly that antibody binding to functional forms of trimeric Env may be sufficient for neutralization, secondly that trimeric Env or proper mimics thereof may make useful vaccine antigens. The principal determinant of neutralization in these studies was antibody avidity, suggesting that the precise target epitope of the antibody was a less important consideration than the tightness of antibody binding to the functional trimer [61]. Moreover, unlike with simple antigens such as V3 peptides for which the antibody dissociation rate was the kinetic parameter correlating best with neutralization [63], on the Env trimer the association rate may also be a significant factor, implying that antibody accessibility to the epitope on the trimer is an important kinetic barrier [60]. This can now be explained by our current structural understanding of Env immune evasion properties relating to steric barriers to antibody-epitope engagement, including glycans and trimer architecture (Section 2). The rate at which an antibody binds its epitope is an important functional feature under conditions of competition with viral receptor engagement and entry, and requires further investigation.

The recent isolation of multiple monoclonal antibodies with potent neutralization activity against a broad range of viral strains (broadly neutralizing antibodies) highlights the fact that Env has conserved, antibody-accessible surfaces on the functional trimer. Some of these antibodies neutralize up to 90% of circulating strains [64], testifying to high conservation of their epitopes. Structural analysis reveals that these antibodies fall into several different epitope-binding clusters, described in more detail elsewhere [29,65,66,67,68,69,70] and summarized in Figure 1. Briefly, these are: (I) the CD4bs; (II) a highly conformational epitope cluster formed from the quaternary folding together of the V1V2 and possibly V3 gp120 loops of at the apex of the trimer; (III) a patch of high mannose glycans alone (2G12 antibody), or together with the underlying protein surface forming glycoprotein epitopes; (IV) a segment of the extramembranal portion of gp41 termed the membrane proximal external region (MPER). Characterization of the atomic structure of the epitopes of these monoclonal antibodies has led to a flurry of activity in the molecular modeling field with the aim of producing conformationally constrained epitope mimetics that could be used to focus B cell responses towards what are otherwise immunorecessive targets. At present this field has produced antigens that share close atomic topology to their native counterparts in the trimer, a spectacular feat of engineering, but these have yet to elicit neutralizing antibodies by immunization [71,72,73,74]. One strategy will therefore be to use such epitope mimetics to prime or boost antibody responses made against native Env trimer structures. This will be discussed in more detail below.

## 4. Preparation of Membrane-Anchored Trimers

**Figure 2:**
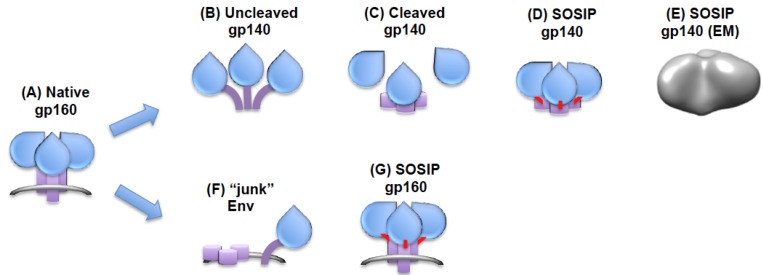
HIV-1 Env trimers for experimental vaccine use. (**A**) Functional, cleaved HIV-1 Env expressed in a viral, infected cell, or VLP membrane; (**B**) Uncleaved soluble gp140 trimer with “open” structure; (**C**) Cleaved gp140 is unstable resulting in subunit dissociation; (**D**) SOSIP gp140 is stabilized by disulfide bonds and maintains a compact “closed” conformation; (**E**) EM reconstruction of BG505 SOSIP gp140 at 24A resolution showing compact globular morphology, from [93] with permission; (**F**) HIV-1 gp160 expressed from infected or transfected cells contains a proportion of so-called “junk” forms that may compete with the native trimeric forms for induction of neutralizing antibodies [79]. These may be at least partially removed by protease treatment [79]; (**G**) Disulfide (SOS)-stabilized membrane-anchored Env trimer [79,90].

There have been numerous attempts to express both membrane-anchored and soluble forms of trimeric Env for immunization. To date none of these attempts have led to induction of potent neutralizing antibodies against highly conserved Env surfaces, but some incremental improvements in neutralization compared to immunization with gp120 have been observed, suggesting that this strategy is worth pursuing [75]. The most straightforward way to produce trimeric Env with a native, functional conformation is to express it in a membrane context. This can be achieved by *in vivo* expression from DNA or from vectors encoding *env*. Obvious advantages of this approach are that the glycoprotein is assembled and expressed in the host cells, therefore being post-translationally processed in a similar manner to the virus during an infection. A downside of this approach is that the amount of Env expressed *in vivo* is unknown, but is likely to be relatively low, generally resulting in weak antibody responses without further protein boosting. Moreover, the antibody evasion mechanisms inbuilt into Env, including endocytosis and the presence of misfolded, uncleaved and subunit dissociated forms will be active in this setting. A more controlled strategy that can potentially deliver greater quantities of antigen is the ex-vivo expression of *env* in the context of Gag, resulting in production of virus-like particles (VLPs) [76,77]. VLPs contain functional trimers and can be purified to high concentration. Membrane-anchored Env produced in high-level expression systems is not free from non-functional forms and contains uncleaved monomer gp160, gp41 stumps and other membrane-anchored “junk” [78,79] (Figure 2). Of particular interest, treatment of membrane Env with protease eliminated most non-native forms whilst preserving native Env, as probed by native gel electrophoresis and neutralizing antibody binding [79,80]. Moreover, soluble gp160 can be liberated from lipid using mild detergent and can be isolated in an intact form, allowing preparation of relatively pure forms of the antigen. Although these approaches raise the challenge of producing sufficiently large quantities of such native antigen for immunization purposes, they are nevertheless promising and deserve further development.

## 5. Preparation of Soluble Trimers

Soluble glycoproteins have obvious advantages over their membrane-anchored counterparts, including ease of manufacture and availability of higher antigen concentrations for immunization. However, the removal of Env from its membrane environment by genetic truncation prior to the transmembrane region (producing a gp140 molecule) results in loss of trimer stability leading to misfolding and gp120-gp41 dissociation (Figure 2). Elimination of the cleavage site between gp120 and gp41 overcomes much of this instability, and many trimeric forms of gp140 have been produced with this feature [75]. Additional stability can be introduced by fusion with an exogenous trimerization motif or by strategic incorporation of a disulfide bond linking gp120 to gp41 in cleaved trimers, termed SOS gp140 [44]. Several studies have compared such trimeric Env antigens with gp120 for immunogenicity and for induction of neutralizing antibodies. The general conclusion from these studies is that first generations of trimeric gp140 yield a small incremental increase in neutralizing antibody elicitation, but to date fail to induce antibodies capable of neutralizing a broad spectrum of HIV-1 circulating strains [81,82,83,84]. 

Although the field lacks an atomic resolution structure of the assembled functional Env trimer, recent antigenic and structural analyses have nevertheless helped define shortcomings in the first generations of soluble trimers, and may provide a path forward to more faithful Env mimics. First, deletion of the gp120-gp41 cleavage site results in a molecule that predictably fails to fold into the mature functional form, and therefore does not antigenically mimic the target antigen on the virus [85,86,87]. The elimination of the gp120-gp41 cleavage site leads to various abnormalities in glycoprotein folding, including the potential for formation of aberrant disulfide bonds between gp120 and gp41 subunits. Although certain surfaces, such as the CD4bs, are well preserved and presented on soluble uncleaved gp140, others such as the quaternary V1V2V3 mAb epitopes are perturbed [88]. Indeed, these antibodies have become extremely useful probes for the conformational integrity of Env, and allow rapid and clear evaluation of trimer folding. The lack of quaternary broadly neutralizing antibodies binding to soluble uncleaved gp140 appears to correspond to structural rearrangements in the trimer that are represented as an “open” conformation in electron tomographic reconstructions of both membrane-anchored and soluble forms of Env [44] (Figure 2). This open conformation can be stabilized in membrane anchored Env by ligation with soluble CD4 or some neutralizing antibodies, and therefore represents the CD4-bound state of the Env trimer, exposing the CRbs and allowing binding of CRbs-specific monoclonal antibodies [44]. In conclusion, uncleaved gp140 represents a form of Env that fails to present some neutralizing antibody epitopes (quaternary epitopes), may present other broadly neutralizing antibody epitopes such as the CD4bs in a manner that fails to mimic the functional trimer, and exposes non-neutralizing antibody epitopes [87]. Inappropriate presentation of the CD4bs will lead to production of antibodies that recognize the surface with an angle of approach that is inconsistent with binding to the functional trimer [37], hence these antibodies will neutralize weakly or not at all. Non-neutralizing surfaces such as cluster-I and -II gp41 epitopes are likely to dominate immunogenicity, and may therefore be deleterious for elicitation of neutralizing antibodies [87]. It therefore seems unlikely that the use of this type of trimeric antigen will yield appropriate neutralizing antibody responses after immunization. As mentioned above, another approach to producing soluble native trimeric Env has been to genetically add a disulfide bond (SOS) that holds the extramembranal portion of gp41 to gp120 post cleavage without obviously disturbing the trimer conformation [89]. When expressed in a membrane bound form these trimers can drive receptor-mediated fusion in the presence of a reducing agent to break the introduced disulfide bond, and so are functional [90]. More recent iterations have introduced a further stabilizing mutation into gp41, producing a construct called SOSIP [91]. Recently described gp140 SOSIP trimers from the BG505 HIV-1 strain lacking most of the MPER have improved solubility [92], a compact and stable structure similar to that of membrane-anchored gp160 as defined by negative stain single particle EM [12,46,93] (Figure 2), and bind all broadly neutralizing antibodies and very few non-neutralizing antibodies [93]. Of particular importance, this antigen binds quaternary epitope-specific mABs, strongly implying correct gp120 folding within the trimer [12,93]. These antigens therefore appear to be very close mimics of the native membrane anchored trimer and the prototype of a new generation of potential vaccine antigens.

## 6. Potential for Success and Current Strategies

Recent advances in the field have increased optimism that pure populations of correctly folded Env trimers can be produced in membrane anchored or soluble forms. This is excellent news as it will, for the first time, allow an evaluation of the structure, antigenicity and immunogenicity of such antigens without contamination from potentially immunodominant, incorrectly folded forms of Env. If immunogenicity could be predicted directly from antigenicity, then these antigens would be expected to elicit antibodies against the surface exposed conserved neutralizing epitopes and not against other non-neutralizing epitopes. However, the relationship between antigenicity immunogenicity is complex and the B cell response to these antigens is unpredictable [27]. In this respect the broadly neutralizing antibodies isolated from HIV-1-infected individuals, which provide proof of principle for the immunogenicity of the target epitopes that elicited them, may not be readily re-elicited by active immunization. Firstly, these antibodies have unusually high levels of somatic hypermutation, most likely driven by reiterative rounds of viral escape by amino acid variation [94]. Such variation will focus B cell responses towards more conserved regions of the antigen, which have unusual epitope structures potentially requiring substantial structural rearrangement of the antibody paratope. Examples of this are: (I) the CD4bs, which as mentioned above imposes stringent steric constraints on the size and shape of the paratope and the angle of approach of the immunoglobulin molecule to the antigen; (II) the MPER that is sandwiched between the lower portion of gp120 and the target cell membrane, driving the selection of antibodies that may bind both protein and lipid [39]. However, lipid binding may not be a prerequisite for MPER antibody function [95], and a recently isolated MPER reactive antibody (10E8) does not require lipid binding, making this epitope a potentially more straightforward target [96]. 

An epitope cluster that may not impose such stringent steric constraints for BCR and antibody recognition is that recognized by the Env quaternary conformation-specific antibodies. This epitope located at the apex of the trimer [12] (Figure 1) appears, at least in models made at molecular-level resolution, not to be subject to the same angle of approach and steric constraints as some other neutralization epitopes [83]. The virus may rely upon the conformation instability of this epitope on the virion to evade efficient B cell recognition. Thus if this epitope cluster was sufficiently stable on the trimer, it may make an effective immunogen for elicitation of this specificity of neutralizing antibody. An approach to eliciting this quaternary conformation Env antibodes may be to completely “fix” the trimer into the appropriate stable conformational state, either using molecular biology to introduce covalent bonds, or by using targeted chemical crosslinking strategies. In this respect, glutaraldehyde cross-linked membrane-bound Env completely stabilizes trimer structure but conserves binding of quaternary conformation antibodies [97].

The immunorecessive nature of particular epitopes may be overcome by priming B cells with epitope mimetics that focus responses to these surfaces. The concept relies upon the presentation of the mimetics by a series of heterologous scaffolds that would each only be used once for immunization thereby focusing B cell responses to the mimetic [74]. This approach is elegant but has its critics, particularly as a stand-alone concept, since re-elicitation of a unique antibody paratope by an isolated epitope is highly improbable [98,99]. However, additional approaches may increase the probability of success. For example, B cell responses initiated by epitope mimetics may be boosted using trimeric Env to drive high-avidity recognition of the native target epitope in its correct molecular environment. If mimetic recognition by naïve BCRs fails, then epitope variants can be engineered to activate naïve B cells and subsequently guide the B cell response towards high-affinity recognition of the target epitope [12,94,100,101]. 

The degree of somatic mutation observed in neutralizing monoclonal antibodies obtained from infected individuals is likely to be very difficult to elicit by active vaccination using a non-persisting antigen. However, the magnitude of mutation observed may be a consequence of B cells reacting to the relentless variation of the target epitope until a non-variable target is arrived at, potentially through inability of the virus to further mutate that region without loss of function. Thus an invariant vaccine antigen may take a short cut to induction of high affinity antibody binding without requiring such a high degree of somatic mutation. Potent new adjuvants, in the form of extrinsic formulations or as co-stimulatory genetic fusions with the antigen may help to achieve efficient affinity maturation by activating T cell help, particularly from T follicular helper cells. Combinations of vector-delivered Env with protein boosting may also increase antibody titers and avidity. 

## 7. Conclusions

The field of trimer-based vaccine antigen development for eliciting neutralizing antibody responses has come a long way over the past 2 decades, and is currently in its most exciting phase. Both membrane-anchored and soluble forms of trimer that are precise mimics of the *in-situ* viral spike have been produced and are currently being characterized for their biophysical, structural, antigenic and immunogenic properties. The next few years will deliver critical information on whether this concept can work, alone or combined with other developing vaccine technologies.
